# Jurypreis des Österreichischen Staatspreises 2018: Zwölf Jahre EFQM (European Foundation for Quality Management) an der Universitäts-Augenklinik Graz

**DOI:** 10.1007/s00347-020-01234-y

**Published:** 2020-10-23

**Authors:** G. Langmann, W. Gliebe, E. Granitz, A. Kohlhofer, S. Reinisch, D. Ivastinovic, A. Wedrich

**Affiliations:** 1 Universitäts-Augenklinik Graz, Medizinische Universität Graz (Med Uni Graz), LKH Universitäts Klinikum Graz, Krankenanstaltengesellschaft der Steiermark (KAGes), Auenbruggerplatz 4, 8010 Graz, Österreich; 2 msi Management Systems International AG, 9490 Vaduz, Fürstentum Liechtenstein

**Keywords:** Qualitätsmanagement, Gesundheitswesen, Committed to Excellence, Recognised for Excellence, Quality Management, Health Care, C2E, R4E

## Abstract

**Fragestellung:**

Kann sich ein Non-Profit-Unternehmen wie eine Universitäts-Augenklinik in Konkurrenz zu Profit-Unternehmen erfolgreich für einen Staatspreis des Bundesministeriums für Innovation und Wirtschaftsstandort in Österreich bewerben.

**Material und Methode:**

Nach erfolgreichen Committed to Excellence (C2E) Assessments 2008/2010 wurde ein 70-seitiger Unternehmensbericht streng nach der EFQM(European Foundation for Quality Management)-Logik (bestehend aus Grundkonzepten, Kriterienmatrix und RADAR-Logik) erarbeitet. Im Assessment 2018 wurde besonderes Augenmerk auf die Entwicklung der Strategie der Universitäts-Augenklinik mit ihren beiden Anteilseignern (Shareholdern), der im Besitz des Landes Steiermark befindlichen Krankenanstalten Gesellschaft (KAGes) und der dem Bundesministerium zugehörigen Medizinischen Universität Graz, gelegt und diese mithilfe der X‑Matrix (nach Hoshin-Kanri) entwickelt und dargestellt.

**Ergebnisse:**

Das Gesamt-Punktescore im Recognised for Excellence(R4E)-Assessment 2018 war 500 bis 550 Punkte, was die Jury zur Vergabe eines Jurypreises des Österreichischen Nationalpreises (Fokus: partizipative Führung) veranlasste. Verbesserungspotenziale waren die Ausrichtung der Kernprozesse auf die Hauptleistungsindikatoren der Universitäts-Augenklinik, die sich aus ihrer Mission, bestehend aus Patient*innen Versorgung, Forschung, Lehre, Ausbildung und Öffentlichkeitsarbeit, ergeben. Die Entwicklung der Strategie mit den Anteilseignern hat sich gegenüber dem R4E-Assessment von 2017 von einem Potenzial zu einer Stärke entwickelt.

**Diskussion:**

Schriftliche und mündliche Rückmeldungen aus einem EFQM-Assessment sind der größte Mehrwert („value-added“) aus diversen Preisbewerbungen für eine Universitäts-Augenklinik sowie allgemein für Wirtschaftsunternehmen.

## Abstract (en)

**Background:**

The European Foundation for Quality Management (EFQM) approach has been successfully implemented in healthcare but reports about EFQM awards at university clinics are lacking so far. Can a non-profit organization, such as a university eye department successfully compete with profit companies from industry for the National Prize of the Federal Ministry for Innovation and Business in Austria?

**Material and methods:**

Following successful committed to excellence (C2E) assessments in 2008 and 2010, a 70-page corporate report was compiled strictly according to the EFQM logic (latest version 2013), consisting of basic concepts, criteria matrix and results/approach/deployment/assessment and refinement (RADAR) logic, which formed the basis for an 1‑day assessment in 2018. Special emphasis was laid on the development and presentation of the strategy by means of the X‑matrix (according to Hoshin-Kanri) between the University Eye Department and its shareholders, the Styrian Hospital Association (KAGes) owned by the Federal State of Styria and the Medical University financed by the Austrian State Federal Ministry.

**Results:**

The total points score in the recognized for excellence (R4E) assessment 2018 was 500–550 points, which prompted the jury to award a jury prize of the Austrian National Prize (focus: participative leadership). Potential for improvement, which needs to be worked on in the near future was the alignment of core processes with the main performance indicators of the University Eye Department, which arises from its mission consisting of patient care, research, teaching, training and public relations activities. The development of the strategy with the shareholders has developed from a potential to a strength compared to the R4E assessment from 2017.

**Conclusion:**

Feedback from the assessor team after the site visit is the greatest added value for a university eye department and/or business enterprise. Improvement measures can be effectively derived following the RADAR logic and lead to a higher quality standard/score and improved performance.

Das EFQM-Modell wurde 1989 durch 14 CEOs der europäischen Wirtschaft entwickelt, erste Erfahrungsberichte im Gesundheitswesen diskutierten die Fragen:

1. inwieweit EFQM gegenüber anderen QM-Systemen (ISO, KTQ) Vorteile bietet,
2. ob ein für die Wirtschaft konzipiertes QM-Modell überhaupt im (universitären) Gesundheitswesen erfolgreich Verwendung finden kann,
3. welche Erkenntnisse eine Universitäts-Augenklinik nach 12-jähriger Beschäftigung (2 Committed to Excellence[C2E]-Auszeichnungen, 2 Recognised for Excellence[R4E]-Auszeichnungen) mit diesem anspruchsvollen Modell kommunizieren kann.

## Hintergrund und Fragestellung

Die Wertigkeit einer Implementierung bzw. die Evidenz der Wirksamkeit eines Qualitätsmanagement(QM)-Systems im Gesundheitssystem wird kontroversiell diskutiert [12].

Während in der Wirtschaft ausgezeichnete nationale oder internationale Unternehmen (Weltmarktführer) in vielen Fällen eine systematische Implementierung eines QM-Systems nachweisen können, sind Berichte über die Implementierung derselben im Gesundheitswesen in den letzten Jahrzehnten selten und meist älteren Datums [2, 3, 5, 6, 13, 15–18, 20].

**Figure 1:**
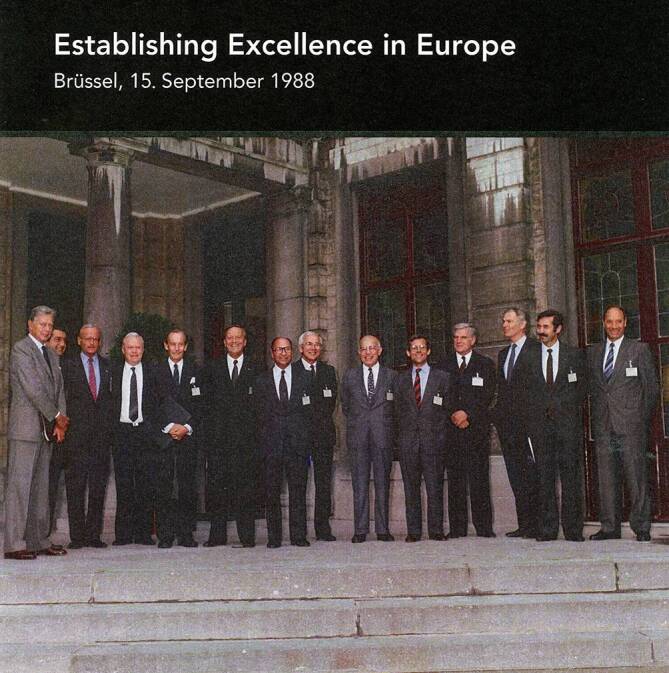

Als wichtigste QM-Systeme haben sich weltweit das ISO 9000 ff [2, 17, 18] sowie das European Foundation for Quality Management(EFQM)-System etabliert. Letzteres wurde 1988 in Brüssel von 14 CEOs exzellenter Unternehmen unter der Schirmherrschaft der Europäischen Union (EU) (Abb. 1), initiiert durch den damaligen Kommissionsvorsitzenden Jaques Delour, gegründet.

Das EFQM-Modell [14, 22] unterscheidet sich vom ISO 9000 ff-System u. a. durch die Vergabe von *nationalen Preisen* (Committed to Excellence – C2E, Recognised for Excellence – R4E, Österreichischer Staatspreis für Unternehmensqualität) und *internationalen* wie dem Europäischen EFQM Excellence Award (EEA) und betont die Stärken eines Unternehmens bei gleichzeitiger Dokumentation der kontinuierlichen Arbeit an Verbesserungspotenzialen.

Im *universitären Gesundheitswesen* waren bislang der Österreichische Staatspreis für Unternehmensqualität und der Europäische Award (EEA) für etwaige Bewerber unerreichbare Ziele.

Diese Arbeit beschreibt jene Schritte/Meilensteine der Universitäts-Augenklinik Graz zur Erreichung des Jurypreises des Österreichischen Staatspreises nach 12-jähriger Projekt‑/Programm-Arbeit.

## Studiendesign und Untersuchungsmethoden

Das EFQM-Modell [4, 14, 15, 22] wurde aufgrund einer gesetzlichen Vorgabe 2006 zur Einführung eines Qualitätsmanagementsystems (Stmk KALG Novelle 2002, Bundes KAG Gesetznovelle 1993) von der Universitäts-Augenklinik und 3 weiteren Organisationseinheiten des LKH Univ. Klinikums Graz ausgewählt und implementiert.

**Figure 2:**
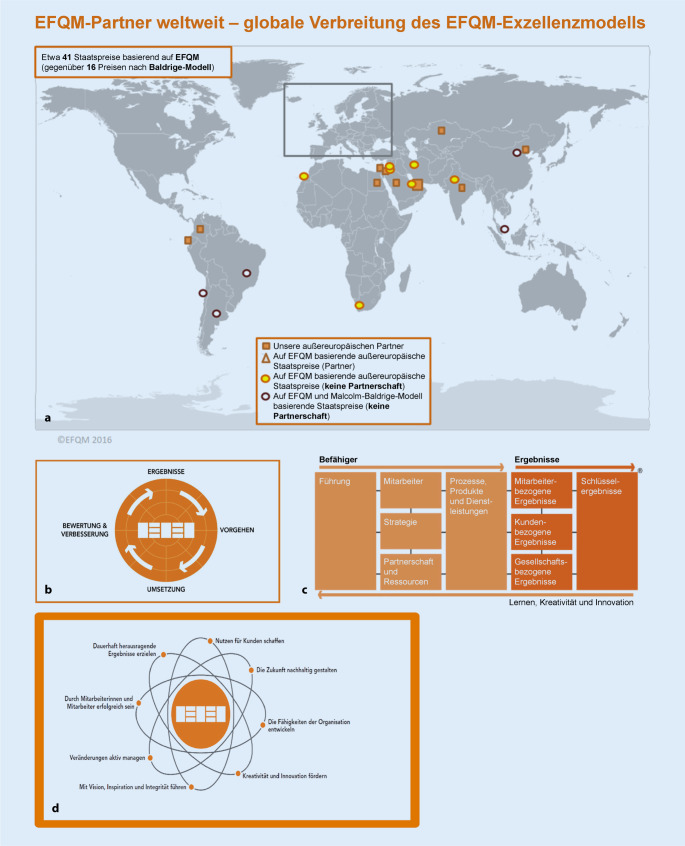

Als Einstieg in die Anerkennungslogik des EFM-Modells (Abb. 2) wurde 2008 und 2010 von der Universitäts-Augenklinik jeweils die Committed to Excellence(C2E)-Auszeichnung angestrebt und auch verliehen.

Im C2E-Assessment wird die Projektarbeit anhand von 3 für die Klinik wesentlichen Projekten beurteilt. Das erste C2E-Projekt der Universitäts Augenklinik Graz wurde 2011 in der Zeitschrift *Der Ophthalmologe* publiziert [8].

Folgende 3 Projekte wurden 2010 für die zweite erfolgreiche C2E-Bewerbung ausgewählt:

1. die Reduktion von ambulanten Patient*innen an der Universitäts-Augenklinik [11],
2. die Entwicklung eines Leitbildes für die Universitäts-Augenklinik [21],
3. die Entwicklung einer Ausbildungsordnung für Assistent*innen in der Ambulanz für Schielen, pädiatrische Ophthalmologie und visuelle Rehabilitation [7, 19].

## R4E-Projektarbeit der Universitäts-Augenklinik

Ein *Redaktionsteam, *bestehend aus der Klinikleitung (die ärztliche und Pflegeleitung) und den Qualitätsbeauftragten der Klinik (ärztlicher Bereich und Pflege), schrieb einen 70-seitigen, nach EFQM-Kriterien strukturierten Unternehmensbericht, der dem Assessor*innen-Team zur theoretischen Beurteilung der Klinik 4 Wochen vor dem Site Visit (Assessment vor Ort) vorgelegt wurde.

Die ersten 5 Kriterien der Kriterienmatrix (die maximal 500 Punkte beim Assessment generieren können) beschreiben die sog. *Befähiger *in einem Unternehmen/der Klinik. Das sind jene Faktoren wie Führung, die Strategie der Klinik, der Bereich Mitarbeitenden sowie die zur Verfügung stehenden Ressourcen und Prozesse der Klinik, die für das Erreichen der Ergebnisse im EFQM-Modell verantwortlich sind.

**Figure 3:**
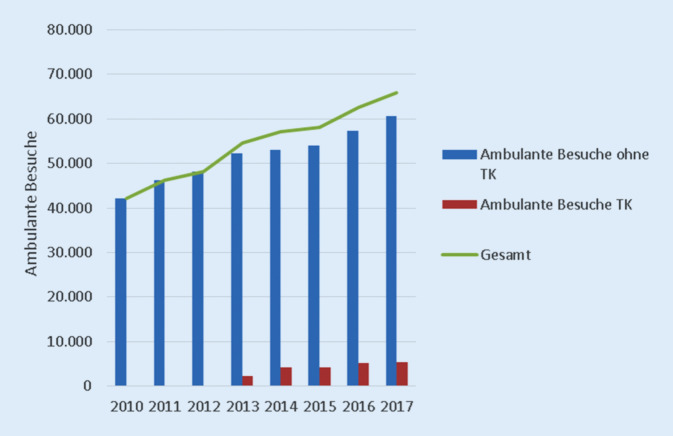

Im *Ergebnisteil* des Modells (mit weiteren maximal 500 zu erreichenden Punkten) finden sich einerseits die Wahrnehmungen, die an der Klinik erhoben wurden, z. B. aus der Patient*innen-Befragung [8] oder der Mitarbeitenden-Befragung in Form von Kennzahlen sowie andererseits die sog. Leistungsindikatoren, das sind Kennzahlen, wie z. B. die Anzahl der ambulanten Patient*innen (Abb. 3). Die Schlüsselindikatoren (im Kriterium 9 zusammengefasst) subsumieren die für die Führung des Unternehmens wesentlichen Kennzahlen, mit der das Unternehmen/die Klinik gesteuert wird. Im Fall der Universitäts-Augenklinik waren dies die Kennzahlen, die sich aus den Missionsfeldern (Patient*innen-Versorgung, studentische Lehre, Forschung, Ausbildung und Öffentlichkeitsarbeit) ableiteten.

**Figure 4:**
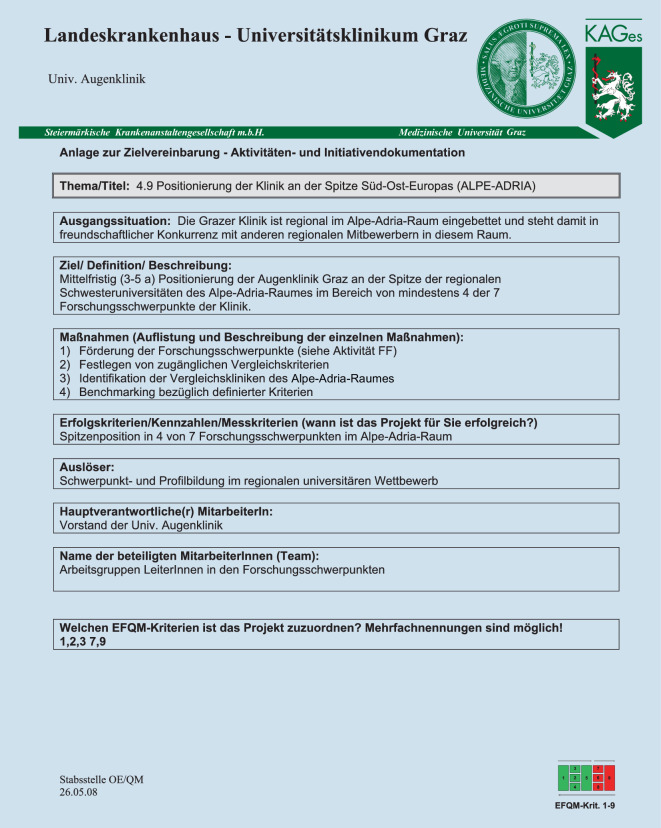

Im *Site Visit* (dem mündlichen Assessment vor Ort) befragte ein Assessoren-Team der Quality Austria ein interdisziplinär zusammengesetztes Team der Klinik und ermittelte letztlich den Gesamtpunkte-Score. Aufgrund des engen Zeitrahmens des Assessments (1 Tag) wurden schriftliche Beweise lediglich stichprobenartig verlangt. Das Assessment passierte auf einer hohen Vertrauensbasis. Beurteilt wird im EFQM v. a., „WIE“ ein Unternehmen seine Ergebnisse generiert, als auch das, „WAS herauskommt“ (z. B. die Ergebnisse). Der positive Ergebnisverlauf über mehrere Jahre und die daraus abgeleiteten Maßnahmen werden mitbeurteilt.

Hohe Punkte-Scores werden erreicht, wenn die Mitarbeiter*innen entsprechend der RADAR-Logik die Planung ihrer Aktivitäten, die Umsetzung bzw. die Kontrolle ihrer Aktivitäten schlüssig nachweisen können. Innovation, Kreativität und kontinuierliches Lernen (z. B. aus einem vorangegangenen Assessment) werden hoch bewertet.

Der finale Assessment-Bericht, aus dem in dieser Publikation die Stärken und Potenziale („vormals Schwächen“ der Klinik) extrahiert wurden, diente der Jury des Österreichischen Staatspreises für die Selektion ihrer Preisträger.

## Ergebnisse

### Assessment 2018

Während die Gesamtpunktezahl bei unserem ersten erfolgreichen R4E-Assessment im *Juni 2017* bei *401 bis 450 Punkten* lag, konnte im zweiten R4E-Assessment im *Juni 2018* eine Steigerung auf *500 bis 550 Punkte* erreicht werden (Tab. 1).

**Table 1:** 

Bewertung (RADAR) (%)	Univ. Augenklinik Graz	Univ. Augenklinik Graz		Staatspreis^a^	Europ. Preis^b^
	Assessment 2017	Assessment 2018		Österreich	EU
1. Führung	51–60	61–70	+	61–70	71–80
2. Strategie	31–40	*61–70*	*++*	51–60	71–80
3. Mitarbeiter*innen	41–50	*61–70*	*++*	61–60	71–80
4. Partner & Ressourcen	41–50	*61–70*	*++*	61–70	71–80
5. Prozesse, Produkte & Dienstleistungen	41–50	51–60	+	61–70	71–80
6. Kunden(Patient*innen)-bezogene Ergebnisse	31–40	41–50	+	51–60	71–80
7. Mitarbeiter*innen-bezogene Ergebnisse	31–40	51–60	++	51–60	61–70
8. Gesellschaftsbezogene Ergebnisse	21–30	*41–50*	*++*	41–50	51–60
9. Schlüsselergebnisse („key results“)	41–50	41–50	–	61–70	71–80
*Gesamtpunkteanzahl*^c^	*401–450*	*500–550*	*–*	*530–680*	*670–750*

^a^Staatspreis 2011–2016

^b^Europäischer Preis (EEA) 2011–2016

^c^Kriterien werden je nach Wertigkeit unterschiedlich gewichtet und mit Korrekturfaktoren multipliziert

Das Hauptaugenmerk in der Projektarbeit zwischen den beiden R4E-Assessments 2017 und 2018 wurde neben der Beurteilung und Bearbeitung aller im Feedbackbericht 2017 angesprochenen Stärken und Potenziale speziell auf die Bearbeitung der *Strategie* der Universitäts-Augenklinik gelegt.

Mithilfe eines externen, international anerkannten EFQM-Coaches und Assessors (einer der Koautoren**)** wurden die Kennzahlen in Form von mittel- bis langfristigen Zielen (Jahreszielen) mit den konkreten Maßnahmen sowie den verantwortlichen Personen mithilfe der x‑Matrix (Hoshin Kanri Strategy Deployment) in Beziehung gesetzt [1].

### Kriterium 1 (Führung)

Die besten Bewertungen erhielt u. a. das *Kriterium 1, die Führung der Klinik,* wobei der *partizipative* Führungsansatz als Begründung für die Zuerkennung des Jurypreises hervorgehoben wurde. Die Universitäts-Augenklinik spielt Vorreiterrollen innerhalb des Universitäts-Klinikums (wie z. B. bei der Erstellung einer gemeinsamen Zielvereinbarung) der Klinik mit ihren beiden Anteilseignern (Shareholdern), der Medizinischen Universität Graz und der Steiermärkischen Krankenanstalten Gesellschaft (KAGes). Die Einführung eines nach dem International Council of Ophthalmology (ICO) ausgerichteten Facharzt-Curriculums [7, 10] wurde des Weiteren als „Role model“ nach EFQM hervorgehoben.

### Kriterium 2 (Strategie)

Die deutliche Weiterentwicklung der strategischen Ausrichtung der Universitäts-Augenklinik bzw. deren Darstellung in Form der X‑Matrix [1] wurde mit 61 bis 70/100 Kriteriumspunkten bewertet und übertraf damit sogar den Durchschnitt aller Bewerber für den Österreichischen Staatspreis (51–60/100).

*Kriterium 4* (Partnerschaften und Ressourcen) wurde mit 61 bis 70/100 Punkten bewertet, wobei sich die Punktezahlen der Universitäts-Augenklinik bereits den Werten des Österreichischen Staatspreises näherten (61–70/100) (Tab. 1).

## Ergebnisteil des EFQM-Kriterienmodells

### Kriterium 6 (Kunden-bezogene Ergebnisse)

Die bereits anlässlich der ersten C2E-Bewerbung 2008 ermittelte hohe Patient*innen-Zufriedenheit konnte auch bei wiederholten Befragungen vonseiten der Stabsstelle Qualitäts‑/Risikomanagement (QM/RM) des Universitäts-Klinikums bestätigt werden [9].

### Kriterium 7 (Mitarbeiter*innen-bezogene Ergebnisse)

Trotz der steigenden Patient*innen-Zahlen (Abb. 3). und der damit einhergehenden erhöhten Anforderungen an die Mitarbeiter*innen der Klinik (Stichwort: hohe Routine-Versorgungsleistung) sind die Ergebnisse der Mitarbeiter*innen-Befragung auf gleichbleibend stabilem Niveau geblieben. Die psychische Belastung am Arbeitsplatz ist lt. Befragung im Vergleich mit dem übrigen Klinikum eher gering ausgefallen.

### Kriterium 9 (Schlüsselergebnisse)

Die Leistung der Klinik wurde in allen Missionsfeldern (Patient*innen, Forschung, Lehre, Ausbildung und Öffentlichkeitsarbeit) mittels zahlreicher Kennzahlen dokumentiert, ein Teil weist positive Trends auf und beweist die anhaltend gute Leistung.

### Verbesserungspotenziale

**Befähigerteil des EFQM-Kriterienmodells (Abb. 2)**
1. *Führung* (Kriterium 1): Eine normbasierte Risikoanalyse entsprechend ISO 31000 und Einbindung in die Prozesse war lt. Feedback-Bericht nur in Ansätzen zu erkennen.
2. *Strategie* (Kriterium 2): Die Darstellung der internen Leistungsfähigkeit in Form einer Kennzahlenmatrix oder eines Dashboards wurde empfohlen. Die Eingliederung der Schlüsselkennzahlen in die Strategie konnte weder im Unternehmensbericht noch im mündlichen Assessment vom Assessoren-Team vollständig nachvollzogen werden und wurde als deutliches Verbesserungspotenzial angeführt.
3. Partner*innen und Ressourcen
  Ein fundierter Partner-Management-Prozess mit Kriterien für die Identifikation von Partner*innen war für die Assessoren nicht klar erkennbar.
4. **Prozesse, Dienstleistungen und Produkte**
  Eine Identifikation von Schlüsselprozessen mit Zuordnung zu den relevanten Interessengruppen und die Prozessmessung mittels Kennzahlen durch Prozessverantwortliche waren für die Assessoren erst im Ansatz zu erkennen.

## Ergebnisteil des EFQM-Kriterienmodells

Auffallend im Ergebnisteil ist eine durchgehend geringere Punktebewertung gegenüber den Befähigern um durchschnittlich 10 bis 15/100 Punkten. Der Grund dafür lag wahrscheinlich darin, dass definierte Ziele bzw. deren Zielerreichung sowie Benchmarks für die Assessoren lediglich im Ansatz sichtbar waren. Eine (zu) hohe Anzahl von Kennzahlen ist im derzeitigen Unternehmensbericht auffallend. Es fehlt der Fokus auf wenige, für die Steuerung der Klinik relevante Kennzahlen bzw. deren Schlüsselprozesse, die sich an einer durchgehenden Strategie orientieren.

## Diskussion

### Partizipativer Führungsstil

Die Universitäts-Augenklinik Graz hat nach 12-jähriger Auseinandersetzung mit dem EFQM Excellence-Modell den *Jurypreis des Österreichischen Staatspreises* 2018 zusammen mit der Universitäts-Klinik für Orthopädie/Traumatologie des LKH Universitäts-Klinikums Graz 2018 erhalten. Dabei wurden der Klinik im EFQM-Wettbewerbsmodell 500 bis 550 Punkte zuerkannt, was der höchstmöglichen Recognised for Excellence(R4E)-Auszeichnung mit 5 Sternen entspricht. Die Anzahl der vergebenen Punkte bewegt sich im österreichischen EFQM-Preis-Niveau. Das Kriterium 3, die Strategie, im Assessment 2017 von den Assessoren noch als deutliches Verbesserungspotenzial (31 bis 40 Punkte) identifiziert, konnte nach Implementierung der x‑Matrix zu einer ausgeprägten Stärke entwickelt werden. Der Punktewert des Kriteriums 2 (Strategie) liegt nun mit 61 bis 70 Punkten bereits im unteren Bereich des europäischen Niveaus (71 bis 80/100 Punkten).

Grundvoraussetzung für die Zuerkennung des Jurypreises war lt. Assessoren-Team u. a. die ständige Weiterentwicklung des *partizipativen Führungsstils*, der bereits 2017 im ersten R4E-Assessment als eine der Stärken der Klinik erwähnt wurde (51 bis 60 Punkte) und 2018 mit 61 bis 70 von 100 möglichen Punkten bewertet wurde.

Das EFQM-Modell unterstützt diesen *partizipativen Führungsansatz *mit abgestufter Einbindung der Mitarbeitenden aller Berufsgruppen in die wesentlichen Entscheidungen der Klinik. Ein hohes Maß an intrinsischer Motivation, die Bereitschaft der Unterstützung aller Klinikziele/des Vorstandes sowie bedingungslose Loyalität der Leistungsträger gegenüber der Klinikführung sind Grundvoraussetzung für einen erfolgreichen partizipativen Führungsansatz, ansonsten ein Scheitern vorprogrammiert ist.

### Partizipativer versus autoritärer Führungsstil

Der partizipative Führungsstil an unserer Klinik wird einerseits durch die *Steuerungsgruppe* (bestehend aus ärztlicher und pflegerischer Führung, Qualitätsbeauftragten, Bereichsverwaltung – verantwortlich für die Finanzen der Klinik und Vertreter der Stabstelle Qualitätsmanagement/Risikomanagement), andererseits durch ein Bekenntnis zur *kollegialen Führung* auf Klinikebene umgesetzt, wonach die ärztliche/pflegerische Führung bzw. deren Mitarbeitende, *gleichberechtigt* zum Wohle der Patient*innen ihre Arbeit verrichten sollen. Dieses Bekenntnis ist auch in der Vision der Klinik [21] festgehalten.

Der *Vorteil der partizipativen Führung* liegt u. a. darin, dass Ideen/Aktivitäten des oberen und mittleren Managements sowie der Mitarbeiter*innen sowohl von ärztlicher als auch pflegerischer Seite gleichberechtigt wahrgenommen und in den Entscheidungsfindungsprozessen berücksichtigt werden.

*Nachteil *eines derartigen Führungsstils ist die relativ hohe Anzahl von Meetings bzw. der hohe zeitliche/personelle Ressourceneinsatz.

Der *Vorteil eines autoritären Führungsstils *im Gegensatz dazu ist, dass Ideen/Visionen rascher umgesetzt werden können, da divergierende Meinungen weniger berücksichtigt werden (müssen) und viele zeit- und personalintensive Treffen wegfallen (*Top-Down-Führungsstil*). Der Ideenpool und die Expertise der Mitarbeiter*innen werden jedoch wenig genutzt.

Vorgesetzte mit einem derartigen Führungsstil sind der *Gefahr* ausgesetzt, dass Fehlentscheidungen wegen der fehlenden oder zu späten Kontrolle zu Fehlentwicklungen mit negativen (Langzeit‑)Folgen führen können.

Der Umstieg von einem autoritären Führungsstil auf einen *partizipativen Führungsstil erfolgte an unserer Klinik in Schritten,* beginnend mit der Einführung regelmäßiger interdisziplinärer Jour fixe der ärztlichen und Pflegeleitungen auf Bereichsebene (Betten-Station, Operationssaal, Ambulanz), in denen bereichsübergreifende Themen diskutiert und gelöst wurden. Das gemeinsame interdisziplinäre Arbeiten an Themen führt zur Entwicklung einer Kultur, die auch im Alltag unmittelbar bei den Patient*innen – z. B. in Form verbesserter Prozesse – ankommt und damit den Zeitaufwand für Besprechungen mehr als kompensiert, was in den positiven Patient*innen-Befragungen wiederholt zum Ausdruck kam.

### Implementierung von C2E und R4E im täglichen Leben einer Klinik

Die große Gefahr bei der Implementierung eines QM-Systems liegt in der Einführung einer teuren *Parallelstruktur,* wenn das seit Jahrzehnten in der Regel an einer Klinik praktizierte Führungs‑/Managementsystem unverändert weitergeführt wird, daneben ein mit hohem zeitlichem/persönlichem Aufwand praktiziertes (EF)QM-System mit kostspieligen Bewerbungen und Coachings für diverse Preise parallel dazu „mitläuft“. Ein solches, rein auf Zuerkennung eines Preises abzielendes Vorgehen erscheint uns aufgrund des beschriebenen hohen Aufwandes und der oft fehlenden Nachhaltigkeit als nicht sinnvoll.

Der *wirkliche Nutzen* bei der Entscheidung zur Implementierung dieses EFQM-Modells liegt in seiner ausgewogenen, in allen Bereichen aufeinander abgestimmten *Ganzheitlichkeit,* verbunden mit dem Anspruch des ständigen Lernens und Verbesserns mithilfe der RADAR-Logik [14, 22], was sich in einer Verbesserung von Ergebnissen niederschlägt und damit auch für übergeordnete Entscheidungsträger interessant ist. Damit ist es auch ein ideales zeitgemäßes Führungsinstrument, das *alle Bereiche der Klinik (EFQM-Hauptkriterien)* – wie z. B. Führung, Strategie, Prozesse und die damit erzielten *Ergebnisse* – berücksichtigt und damit für Leiter*innen von Klinken auch als Leitfaden in der täglichen Führungsarbeit dienen kann.

Als großer Nutzen erwies sich die interdisziplinäre Erarbeitung des „*Unternehmensberichtes*“ der Klinik, in der alle EFQM-Kriterien und Subkriterien, bezogen auf die Klinik, betrachtet wurden. Damit verschafften sich die Mitglieder der Führungsebene nicht nur eine Gesamtübersicht über die Klinik und ihre Bereiche – ein ideales Instrument auch für die Neuübernahme einer Klinik –, sondern identifizierten einige Potenziale/z. B. Fehlen eindeutiger Regelungen und Prozesse, die interdisziplinär aufgearbeitet und verbessert wurden.

Im Alltag ist die Implementierung des EFQM für die Mitarbeiter*innen *oft nicht als Qualitätsmanagement bewusst*, da sich die Unternehmenskultur über Jahre/Jahrzehnte stetig verändert hat. So werden z. B. jedes Jahr die *strategischen Ziele der Klinik interdisziplinär* formuliert, strukturiert abgearbeitet, und die Zielerreichung wird reflektiert. Diese Ziele fließen bei den jährlichen Zielvereinbarungen mit der Universität und dem Krankenanstaltenträger ein, bei der z. B. zu erfüllende OP-Zahlen (Einnahmen) als Schlüsselergebnis, aber auch Themen wie Infrastrukturerweiterungen, Personalressourcen oder Maßnahmen zur Erhöhung der Patient*innen-Zufriedenheit bearbeitet werden.

Eine neu etablierte *Steuerungsgruppe*, bestehend aus den Führungskräften aller Berufsgruppen unserer Klinik, *kontrolliert in quartalsmäßigen Abständen* neue sowie bereits existente/gelebte Aktivitäten der Klinik (Abb. 4), einmal jährlich wird die Gesamtstrategie der Klinik reflektiert. Diese Initiativen/Aktivitäten, schriftlich in einem besonderen Formular der Stabsstelle QM/RM der KAGes genau dokumentiert, sind auf operativer Ebene ein wesentlicher Bestand des gelebten partizipativen Führungsstils und entscheidender Teil der Strategie der Universitäts-Augenklinik.

Das EFQM bietet mit den *Committed to Excellence(C2E)-Assessments* einen idealen Einstieg in die EFQM (Wettbewerbs)-Logik, wobei Projektmanagement und Prozessmanagement anhand von 3 mittels Priorisierungsmatrix selektionierten Projekten und deren operative Umsetzung u. a. mittels Meetingprotokollen, Zahlen und Fakten von unabhängigen Assessoren der Quality Austria beurteilt wurden.

Die Universitäts-Augenklinik Graz hat *zweimal diese C2E-Assessments* [10, 11, 19, 21] mit unterschiedlichen Teams mit für die Klinik zum jeweiligen Zeitpunkt wichtigen Projekten durchlaufen und anschließend das über das Feedback Gelernte in vielen weiteren Projekten über Jahre hindurch bis hin zur Erstellung eines „*Unternehmungsberichtes*“ weitergeführt. Letztendlich wurde der Bericht bei der Quality Austria zum Staatspreis eingereicht, um auch über eine Evaluierung durch externe, in der Beurteilung von Unternehmungsführung erfahrene Assessor*innen Rückmeldungen zur Verbesserung zu erhalten.

Damit die EFQM-Philosophie auch nach Ausscheiden von erfahrenden (EFQM) Kolleg*innen weiterlebt, werden neue Vertreter der Qualitätsbeauftragen frühzeitig bestimmt, in die EFQM-Meetings eingeladen und durch Ausbildungen der Quality Austria auf hohem Niveau auf ihre neuen Funktionen vorbereitet. Neuen Assistent*innen der Klinik werden im Rahmen von praktischen Assistent*innen-Fortbildungen Grundbegriffe des EFQM vorgestellt.

Vision und Mission werden im Rahmen der *Mitarbeitergespräche* vom Vorstand der Klinik und der Pflegeleitung abgefragt und diskutiert. Die *EFQM-Meilensteine/Auszeichnungen* der Klinik werden in Form von Posterpräsentationen bei nationalen und internationalen Kongressen präsentiert, diese Poster werden nach Rückkehr von diversen Kongressen an der Klinik dauerpräsentiert.

### Risiken und Grenzen eines QM-Prozesses

Bei der Implementierung eines (EF)QM-Systems sollte sich jedes Mitglied eines QM-Teams/bzw. die Klinikführung einen besonderen Quotienten im Hinblick auf Verhalten der Mitarbeiter*innen vor Augen halten, um nicht unrealistische Ziele zu setzen bzw. frühzeitigen Frustrationen zu erliegen.

Der* Quotient 20:60:20* bedeutet, dass zum Zeitpunkt der Implementierung eines QM-Systems zumindest *20* *% der Mitarbeitenden* von einer (EF)QM-Implementierung überzeugt, idealerweise sogar begeistert sind und diese Gruppe die neue (QM-)Bewegung hauptverantwortlich tragen soll.

In einer Warteposition verharren *60* *% der Mitarbeitenden der Klinik *(und werden sich zum gegebenen Zeitpunkt entweder den ersten 20 %, den letzten 20 % zuwenden oder in der ursprünglichen Position der Mehrheit, den 60 %, verharren). Diese Mehrheit der Mitarbeitenden beobachtet den Fortschritt der Einführung eines QM-Systems und entscheidet sich im Verlauf der (EF)QM-Implementierung, ob sie Teil der EFQM-Bewegung werden will.

Die restlichen *20* *% sind Skeptiker/Gegner* eines (EF)QM-Systems von der ersten Stunde an und werden es, wie auch selbst an unserer Klinik erfahren, immer bleiben. Die Herausforderung für die Unternehmensführung bleibt, diesen Skeptikern/Gegnern nicht zu viel Gewicht/Beeinflussungspotenzial zu geben und ihnen gegenüber mit Geduld und Sachlichkeit zu agieren.

### Kosten und Zeitdruck in der Krankenversorgung

Der universitäre Alltag ist aktuell geprägt von steigenden Patient*innen-Bedürfnissen und -Zahlen, Kostensteigerungen bei Diagnostik und Therapie bei gleichbleibenden oder sogar sinkenden (Personal‑)Ressourcen sowie zögerlichen Finanzierungen der universitären Einrichtungen, was letztlich zu mangelnder Zeit für Forschung, Lehre sowie Ausbildung führt.

In dieser Situation ist kein QM-System ein unmittelbares, rasch wirksames Heilmittel, da es primär auf die optimale Nutzung des vorgegebenen Handlungsrahmens und der bestehenden Ressourcen abzielt.

Dennoch macht die Implementierung eines (EF)QM-Systems Sinn. Erst wenn die bestehenden Ressourcen optimal ausgenutzt sind, Prozesse optimiert werden, (Patient*innen‑)Zahlen die Notwendigkeit einer Personalerhöhung eindeutig belegen, können ernst zu nehmende Verhandlungen mit den Anstaltenträgern auf Augenhöhe mit der Perspektive eines positiven Ausgangs geführt werden.

### Qualitätssicherung

Qualitätssicherung ist die erste, wichtige Stufe eines QM-Systems mit einem Business Excellence-Ansatz, wie ihn das EFQM repräsentiert. Mit Qualitätssicherung wird einerseits der Notwendigkeit der verpflichtenden, ärztlichen Dokumentation, andererseits der Erfassung von validen Daten für hochwertige Publikationen entsprochen.

Dies setzt jedoch *abgestimmte Prozesse, entsprechendes Zeitbudget* bzw. *ausreichend Personal* voraus, was an einer Universitäts-Klinik mit den Missionsfeldern Patient*innen-Versorgung, Lehre und Forschung und Ausbildung eine stetig präsente, große Herausforderung darstellt.

### Strategie der Universitäts-Augenklinik

Eines der größten Herausforderungen bei der Entwicklung der Strategie der Universitäts-Augenklinik waren die divergierenden Strategien ihrer beiden Shareholder (Anteilseigener) bzw. Interessenpartner (Stakeholder), der Krankenanstalten Gesellschaft der Steiermark (KAGes) und der Medizinischen Universität Graz (Med Uni Graz) bzw. deren Geldgeber, dem Land Steiermark und dem Österreichischen Nationalstaat, die historisch gesehen zwar ähnliche Leitbilder (einschließlich Vision, Mission) publiziert hatten, in realiter jedoch unterschiedliche Strategien (z. B. unterschiedliche Dienstverträge, divergierende Schwerpunkte) verfolgen. Die KAGes legt ihren Fokus auf die Patient*innen-Versorgung im Sinn eines Versorgungsauftrages des Landes Steiermark, unterstützt jedoch auch die Schwerpunkte der Medizinischen Universität. Die Agenden Forschung und Lehre (das 2. und 3. Missionsfeld der Universitäts-Augenklinik) werden schwerpunktmäßig von der Medizinischen Universität (Med Uni Graz) vor dem Hintergrund der Patient*innen-Versorgung vertreten. Die Universitäts-Augenklinik hat neben diesen 3 Missionsfeldern ihrer Shareholder/Stakeholder noch die Missionsfelder Ausbildung und Öffentlichkeitsarbeit hinzugefügt, da diese ihrer Einschätzung nach in der Mission von Med Uni Graz und KAGes (Krankenanstaltengesellschaft der Steiermark) zu gering repräsentiert waren.

Die Universitäts-Augenklinik hat mit einem erfolgreichen Projekt im Rahmen ihres Committed to Excellence(C2E)-Preises 2008 gezeigt, dass eine gemeinsame, abgestimmte Zielvereinbarung zwischen KAGes, Med Uni Graz und einer ihrer Organisationseinheiten, namentlich der Universitäts-Augenklinik, in einem sehr engen, durch das EFQM C2E-Assessment vorgegebenen Zeitrahmen bereits 2008 innerhalb von 6 Monaten umgesetzt werden konnte.

Dieses C2E-Pilotprojekt der Universitäts-Augenklinik „*Gemeinsame Zielvereinbarung mit beiden Stakeholdern*“ wurde kurz nach dem erfolgreichen Assessment 2008 auf alle Kliniken/Abteilungen des LKH Univ. Klinikums Graz ausgerollt und weiterentwickelt. Damit sollen auf diese Weise die unterschiedlichen Schwerpunkte Krankenversorgung auf der einen Seite, Lehre und Forschung auf der anderen Seite, gleichberechtigt in die strategischen Überlegungen und letztlich in die schriftlichen Zielvereinbarungen der Organisationseinheiten einfließen. Die jahrelange Entwicklung der Ergebnisse, basierend auf Kennzahlen in allen relevanten Bereichen, wie z. B. Zufriedenheit der Studierenden, Patient*innen, Publikationsleistungen, OP-Zahlen, kann mit dem EFQM-Modell, das ursprünglich für die Industrie entwickelt wurde, besser mit ihren Stakeholdern/Shareholdern kommuniziert werden.

### Gemeinsame Strategie des LKH Univ. Klinikums Graz

Je weiter Vision und Mission der einzelnen Organisationseinheiten (Universitäts-Kliniken, Abteilungen, Institute) bzw. ihrer Shareholder/Shakeholder (Land, Bund) voneinander abweichen, desto eher konkurrieren diese divergierenden strategischen Ausrichtungen und schwächen die Umsetzung von Mission als auch Vision des Gesamtklinikums ab, im ungünstigen Fall herrscht Stillstand.

Die Vision hätte theoretisch dann ihre größte Wirkung, wenn sie mit der Mission ihrer Träger übereinstimmt bzw. sich der strategischen Ausrichtung der Shareholder/Stakeholder annähert. So ist die Mission von J.F. Kennedy für die USA, erstmals eine bemannte Mondlandung zu erzielen (im vergangenen Jahr 50-Jahre-Jubiläum), die stärkste je formulierte Vision. Nachdem J.F. Kennedy hier einerseits die Vision vorgibt, andererseits auch den Auftrag dazu (die Mission) und auch die Geldmittel zur Verfügung stellen kann, konvergieren hier Vision und Mission maximal. Das Resultat ist Geschichte.

Zur Entwicklung einer gemeinsamen Strategie im gesamten Universitäts-Klinikum Graz wurden bereits 2011 gemeinsame Arbeitspakete zwischen Med Uni Graz und KAGes erarbeitet, die z. T. schon in die Routine umgesetzt worden sind. Unter Anwendung des EFQM-Ansatzes wurden bereits wertvolle Initiativen am LKH Universitäts-Klinikum auf höchster Managementebene gesetzt (Anstaltsleitung des LKH Universitäts-Klinikums – Committed to Excellence (C2E), Department für Endokrinologie der Medizinischen Universitätsklinik Graz – Recognised for Excellence (R4E), KAGes Führung Management (Finalist beim Österreichischen Nationalpreis 2017), Universitätsklinik für Orthopädie/Traumatologie (Jurypreis beim Österreichischen Staatspreis 2018).

Eine gemeinsame Strategie zwischen den Anteilseignern (Shareholdern) bzw. Stakeholdern (Interessenpartnern) wie KAGes und Med Uni Graz im Universitäts-Klinikum mit Einbindung aller Organisationseinheiten ist eine *seit Jahrzehnten formulierte/geforderte Vision mit ungeahntem Effektivitäts- bzw. Effizienzpotenzial.* Eine *Top-Down-Implementierung eines Business Excellence-Systems wie dem EFQM-Modell auf höchster Managementebene* mit allen Trägern unserer Organisationseinheiten könnte aufgrund erfolgreicher Vorarbeiten im EFQM-Ansatz einen entscheidenden Beitrag für eine erfolgreiche Finalisierung einer gemeinsamen Grundstrategie zwischen Krankenanstalten Gesellschaft (KAGes), Medizinischer Universität und deren Shareholdern, dem Österreichischen Nationalstaat, dem Land Steiermark sowie den Organisationseinheiten am Universitäts-Klinikum Graz leisten.

## Fazit für die Praxis

- Die Universitäts-Augenklinik Graz konnte 2018 nach 12-jähriger Beschäftigung mit dem EFQM-Modell in Konkurrenz zu exzellenten Wirtschaftsunternehmen einen Jurypreis im Zuge der Verleihung des Österreichischen Staatspreises erlangen.
- Im Gegensatz zur ISO-Matrix, bei der Qualitätsnachweise gefordert werden, jedoch eine Weiterentwicklung im Sinn höherer Punkte nicht honoriert wird, handelt es sich beim EFQM-Modell um ein Wettbewerbs- bzw. Reifegradmodell, das die unterschiedliche Implementierung von Qualität in Form von Assessments beurteilt und dafür Punkte bzw. Preise (national, international) vergibt.
- Grundvoraussetzung für die Erlangung des Jurypreises waren 2 Committed to Excellence(C2E)-Preise (2008, 2010) bzw. eine erste Recognised for Excellence(R4E)-Auszeichnung 2017 sowie eine ständige Verbesserung der Potenziale, wie z. B. der Strategie der Klinik mit ihren beiden Anteilseignern (Shareholdern).
